# Integrated bioinformatics analysis of aberrantly-methylated differentially-expressed genes and pathways in age-related macular degeneration

**DOI:** 10.1186/s12886-020-01392-2

**Published:** 2020-03-24

**Authors:** Yinchen Shen, Mo Li, Kun Liu, Xiaoyin Xu, Shaopin Zhu, Ning Wang, Wenke Guo, Qianqian Zhao, Ping Lu, Fudong Yu, Xun Xu

**Affiliations:** 1 Department of Ophthalmology, Shanghai General Hospital, School of Medicine, Shanghai Jiaotong University, Shanghai, 200080 People’s Republic of China; 2 National Clinical Research Center for Eye Diseases, Shanghai Key Laboratory of Ocular Fundus Diseases, Shanghai Engineering Center for Visual Science and Photomedicine, Shanghai Engineering Center for Precise Diagnosis and Treatment of Eye Diseases, Shanghai, China; 3 Bio-X Institutes, Key Laboratory for the Genetics of Developmental and Neuropsychiatric Disorders (Ministry of Education), Shanghai Jiao Tong University, Shanghai, China; 4 NHC Key Lab. of Reproduction Regulation (Shanghai Institute of Planned Parenthood Research), Fudan University, Shanghai, China

**Keywords:** Methylation, Gene expression, Age-related macular degeneration, Choroidal neovascularization, Bioinformatics analysis

## Abstract

**Background:**

Age-related macular degeneration (AMD) represents the leading cause of visual impairment in the aging population. The goal of this study was to identify aberrantly-methylated, differentially-expressed genes (MDEGs) in AMD and explore the involved pathways via integrated bioinformatics analysis.

**Methods:**

Data from expression profile GSE29801 and methylation profile GSE102952 were obtained from the Gene Expression Omnibus database. We analyzed differentially-methylated genes and differentially-expressed genes using R software. Functional enrichment and protein–protein interaction (PPI) network analysis were performed using the R package and Search Tool for the Retrieval of Interacting Genes online database. Hub genes were identified using Cytoscape.

**Results:**

In total, 827 and 592 genes showed high and low expression, respectively, in GSE29801; 4117 hyper-methylated genes and 511 hypo-methylated genes were detected in GSE102952. Based on overlap, we categorized 153 genes as hyper-methylated, low-expression genes (Hyper-LGs) and 24 genes as hypo-methylated, high-expression genes (Hypo-HGs). Four Hyper-LGs (*CKB*, *PPP3CA*, *TGFB2*, *SOCS2*) overlapped with AMD risk genes in the Public Health Genomics and Precision Health Knowledge Base. KEGG pathway enrichment analysis indicated that Hypo-HGs were enriched in the calcium signaling pathway, whereas Hyper-LGs were enriched in sphingolipid metabolism. In GO analysis, Hypo-HGs were enriched in fibroblast migration, membrane raft, and coenzyme binding, among others. Hyper-LGs were enriched in mRNA transport, nuclear speck, and DNA binding, among others. In PPI network analysis, 23 nodes and two edges were established from Hypo-HGs, and 151 nodes and 73 edges were established from Hyper-LGs. Hub genes (*DHX9*, *MAPT*, *PAX6*) showed the greatest overlap.

**Conclusion:**

This study revealed potentially aberrantly MDEGs and pathways in AMD, which might improve the understanding of this disease.

## Background

Age-related macular degeneration (AMD) is the leading cause of adult blindness in developed countries, particularly in those over 55 years of age. Worldwide, this condition accounts for 7% of cases of blindness [1, 2] and is expected to affect 288 million people by the year 2040 [3]. AMD first appears as drusen (dry AMD) and advances to late AMD (wet AMD) characterized by choroidal neovascularization (CNV) [4]. Drusen is composed of lipoproteinaceous deposits and acellular debris [5]. CNV involves the growth of new abnormal blood vessels originating from the choroid through a break in the Bruch’s membrane, which then invade the subretinal pigment epithelium or sub-retinal space, resulting in severe vision loss [6, 7]. AMD affects central fine vision, significantly impairs a patient’s ability to drive, read, and recognize faces, and greatly affects quality of life [8]. As for wet-AMD (i.e. advanced stages), anti-vascular endothelial growth factor (anti-VEGF) therapy was shown to be effective and has become the first choice for the treatment of CNV [9]. However, anti-VEGF therapy requires repeated intra-vitreal injections, which are associated with a risk of infection and treatment burden for both the patients and the ophthalmologists [10]. Moreover, some patients have poor response to the drugs after a long-term treatment [11]. Apart from anti-VEGF drugs, some other therapies have also impacted macular disease treatment and showed their effectiveness, such as dexamethasone implant [12–14].

Previous studies indicated that many environmental factors are associated with an increased risk of AMD, such as age, race, smoking, obesity, and hypertension [15, 16]. Additionally, genetic factors are regarded as important for the initiation and progression of AMD [17, 18]. Comparing to tumor tissue samples, ocular fundus tissue samples (i.e. retina and choroid) of patients with AMD are quite difficult to obtain in real world. The difficulties to obtain human fundus tissues restricted our understanding of this blinding disease. Over the past few years, most genetic studies on AMD were case-control genome-wide association studies (GWASs) of single-nucleotide polymorphisms in the patients’ peripheral blood [19–21], which were valuable but provided limited information.

With the rapid development of gene assay technology, studies of disease pathogenesis are no longer limited to gene deletions, gene mutations, and gene insertions, among other changes. Microarrays based on high-throughput platforms are useful and efficient tools to search for meaningful genes and epigenetic alterations for the identification of diagnostic or prognostic biomarkers [22]. To better explore the molecular mechanism underlying AMD, it is necessary to conduct full transcriptome analysis at the tissue level; however, as mentioned previously herein, obtaining ocular tissue samples is difficult. Compared to retina or choroid tissue, blood samples are relatively easy to obtain from patients with AMD. Therefore, it would be helpful to evaluate gene expression in the ocular tissue of patients with AMD as biomarkers in the blood. In the present study, data from gene expression profiling microarrays of human retinal and choroidal samples from the Iowa and Oregon cohort AMD and control donors (GSE29801: https://www.ncbi.nlm.nih.gov/geo/query/acc.cgi?acc=GSE29801) [23], as well as gene methylation profiling microarrays of the peripheral blood of subjects with AMD (GSE102952: https://www.ncbi.nlm.nih.gov/geo/query/acc.cgi?acc=GSE102952), were integrated and analyzed using a series of bioinformatics tools. More precise screening results were obtained by overlapping these two AMD data sets. Few studies have attempted to combine gene expression profile microarrays and gene methylation profile microarrays to understand the development of AMD. The introduction of DNA methylation characteristics in the blood is useful to understand the characteristics of AMD disease at the tissue level.

In the present study, data from gene expression profiling microarrays and gene methylation profiling microarrays were integrated and analyzed. Functional enrichment and protein–protein interaction (PPI) network analyses of screened genes were performed using the R package “clusterProfiler” and Search Tool for the Retrieval of Interacting Genes (STRING) online database. We identified methylated genes in the peripheral blood, which might be useful as biomarkers for the precise diagnosis and treatment of AMD.

## Methods

The need for ethics approval was waived by the ethics committee of Shanghai General Hospital, Shanghai Jiao Tong University School of Medicine, Shanghai, China (the document is attached as an additional file).

### Microarray data information

We identified methylated, differentially-expressed genes (MDEGs) between AMD and control samples by analyzing mRNA microarray and methylation profiling datasets. In this study, a gene expression profiling dataset (GSE29801: https://www.ncbi.nlm.nih.gov/geo/query/acc.cgi?acc=GSE29801) and gene methylation dataset (GSE102952: https://www.ncbi.nlm.nih.gov/geo/query/acc.cgi?acc=GSE102952) were downloaded from the Gene Expression Omnibus (https://www.ncbi. nlm.nih.gov/geo/) of the National Center for Biotechnology Information. The gene expression profiling data were based on the mRNA from the macular regions of human donor eyes from the retina and retinal pigment epithelium (RPE)-choroids. The gene methylation microarray data were assessed using genome-wide DNA methylation profiling of peripheral blood.

Microarray data from GSE29801 included 177 samples from the macular or extramacular regions of human donor eye RPE-choroids and 118 samples from the macular or extramacular region of human donor retinas with no reported ocular disease, with possible preclinical AMD or AMD [23]. RPE-choroid and retinal samples were isolated from human donor eyes obtained from the University of Iowa (GSH) and Lions Eye Bank of Oregon. The Iowa eyes were selected from a well-characterized repository derived from more than 3900 donors. The Oregon eyes were generally classified as AMD based on medical histories confirmed by ophthalmological records [23, 24]. Global transcriptome profiling was carried out using the Agilent Whole Human Genome 4 × 44 K in situ oligonucleotide array platform (G4112F, Agilent Technologies, Santa Clara, CA, USA). After removing redundant data, the microarray data of 41 patients with AMD and 42 normal controls were included in our analysis [23]. First, the authors removed redundant data and selected the subjects who had both macular retina and macular RPE-choroid records. Such selection criteria were implemented due to the fact that AMD mainly affects central vision acuity (i.e. the central macular area). Second, we compared the gene expression levels of macular retina and macular RPE-choroid, then, we chose the higher one of them to conduct the further bioinformatics analysis.

For the gene methylation microarray data, Oliver et al. previously performed genome-wide DNA methylation profiling of blood from nine patients with AMD and nine controls based on the GPL13534 Illumina HumanMethylation 450 BeadChip platform (HumanMethylation450_15017482, San Diego, CA, USA), which covers approximately 450,000 CpG sites in different gene regions including the transcription start sites 1500 and 200, 5′ untranslated region, 1st exon, body, and 3′ untranslated region. The authors generously shared their original data online for public use (GSE102952: https://www.ncbi.nlm.nih.gov/geo/query/acc.cgi?acc=GSE102952). The methylation profile data of all nine patients with AMD and nine normal controls were included in this analysis.

### Data processing to identify differentially-expressed genes (DEGs) and differentially-methylated genes (DMGs)

We used the Limma package in R software (version 3.4.2; Bell Laboratories, formerly AT&T, now Lucent Technologies, Murray Hill, NJ, USA) to analyze GSE29801 and GSE102952 to identify DEGs and DMGs. *P* < 0.05 was regarded as statistically significant. For DEGs, we set the cut-off standard as *P* < 0.05 and the absolute value of the log (fold-change) > the median (fold-change). For DMGs, we set the cut-off criteria as *P* < 0.05 and absolute value of log (fold-change) > 3/4 sort [summary log (fold-change)]. For further analysis, hypomethylation-high expression genes (Hypo-HGs) were obtained by overlapping hypomethylated and upregulated genes; hypermethylation-low expression genes (Hyper-LGs) were obtained by overlapping hypermethylated and downregulated genes.

### Functional enrichment analysis

Gene ontology (GO) and Kyoto Encyclopedia of Genes and Genomes (KEGG) pathway enrichment analyses were performed for the selected genes (Hypo-HGs and Hyper-LGs), and the enrichment results were illustrated using the R package “clusterProfiler”. This package can be used to extract biological meaning from large gene lists. We performed GO term enrichment analysis under the following three sub-ontologies: biological process (BP), molecular function (MF), and cellular component (CC). The cut-off criterion of significantly-enriched KEGG pathways was *P* < 0.05.

### Comprehensive PPI network

Determination of the comprehensive PPI network is important to detect the molecular mechanisms of AMD. In this study, we used the online STRING (version 11.0) tool to construct the network of Hypo-HGs and Hyper-LGs. STRING is an online database used to predict PPIs, which are essential to interpret the molecular mechanisms of key cellular activities in AMD. The cut-off standard was defined as an interaction score of 0.4. The results were visualized in Cytoscape software (version 3.5.1). Hub genes were defined as the top three genes that appeared most frequently according to all cytoHubba ranking methods using Cytoscape software. Subsequently, the Molecular Complex Detection (MCODE) algorithm in Cytoscape software was used to screen the modules. An MCODE score > 3 and node number > 3 were used as the criteria to define a module.

## Results

### Identification of DEGs and DMGs in AMD

To identify DEGs or DMGs, we used the expression profile from GSE29801 (containing RPE-choroid and retina tissue samples from 41 patients with AMD and 42 normal samples) and the methylation profile from GSE102952 (containing peripheral blood samples of nine AMD patients and nine normal controls) after data preprocessing and quality assessment using R software. We identified 827 high-expression genes and 592 low-expression genes and 4117 hypermethylated genes and 511 hypomethylated genes. The top 100 most significant DEGs of GSE29801 are shown in Table S1, and the top 100 most significant DMGs of GSE102952 are shown in Table S2.

### Identification of aberrantly-methylated DEGs in AMD

**Fig. 1:**
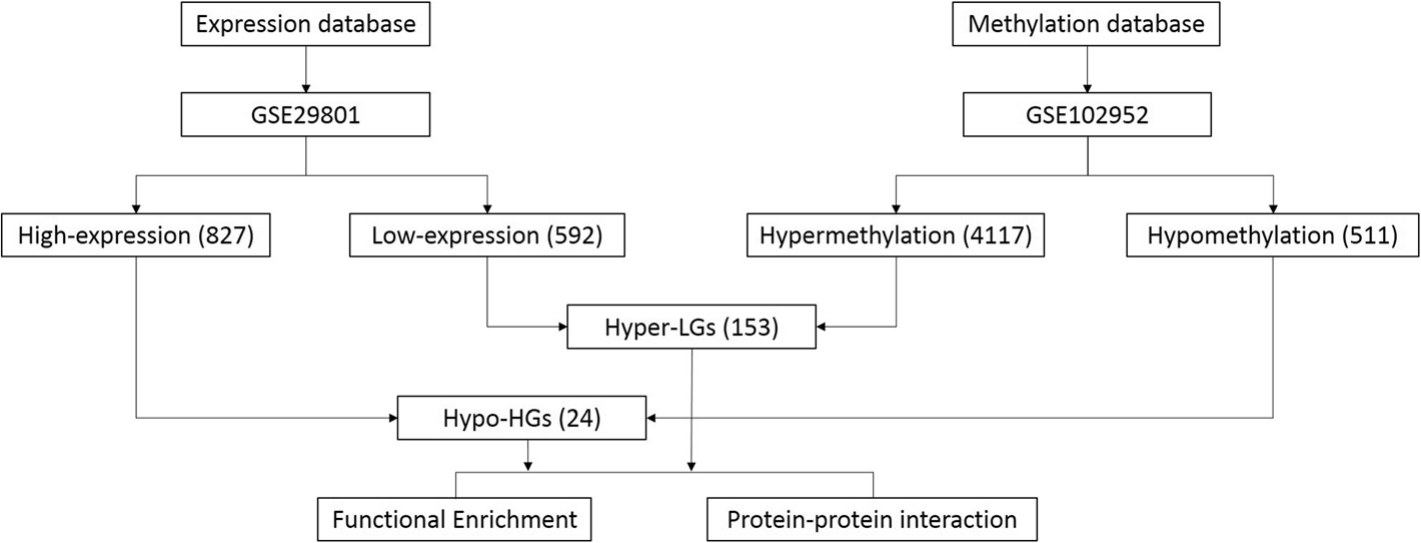
The flowchart of this study. The data of expression profiling GSE29801 and methylation profiling GSE102952 were obtained from Gene Expression Omnibus (GEO) database. As a result, we identified 827 high-expression genes and 592 low expression genes; 4117 hypermethylated genes and 511 hypomethylated genes. To further explore the aberrantly methylated differentially expressed genes, Hypo-HGs were obtained by overlapping hypomethylation and up-regulated genes; Hyper-LGs were obtained by overlapping hypermethylation and down-regulated genes. In total, we identified 24 Hypo-HGs and 153 Hyper-LGs. Then, functional enrichment analysis and protein–protein interaction (PPI) network analysis of screened genes were performed

**Fig. 2:**
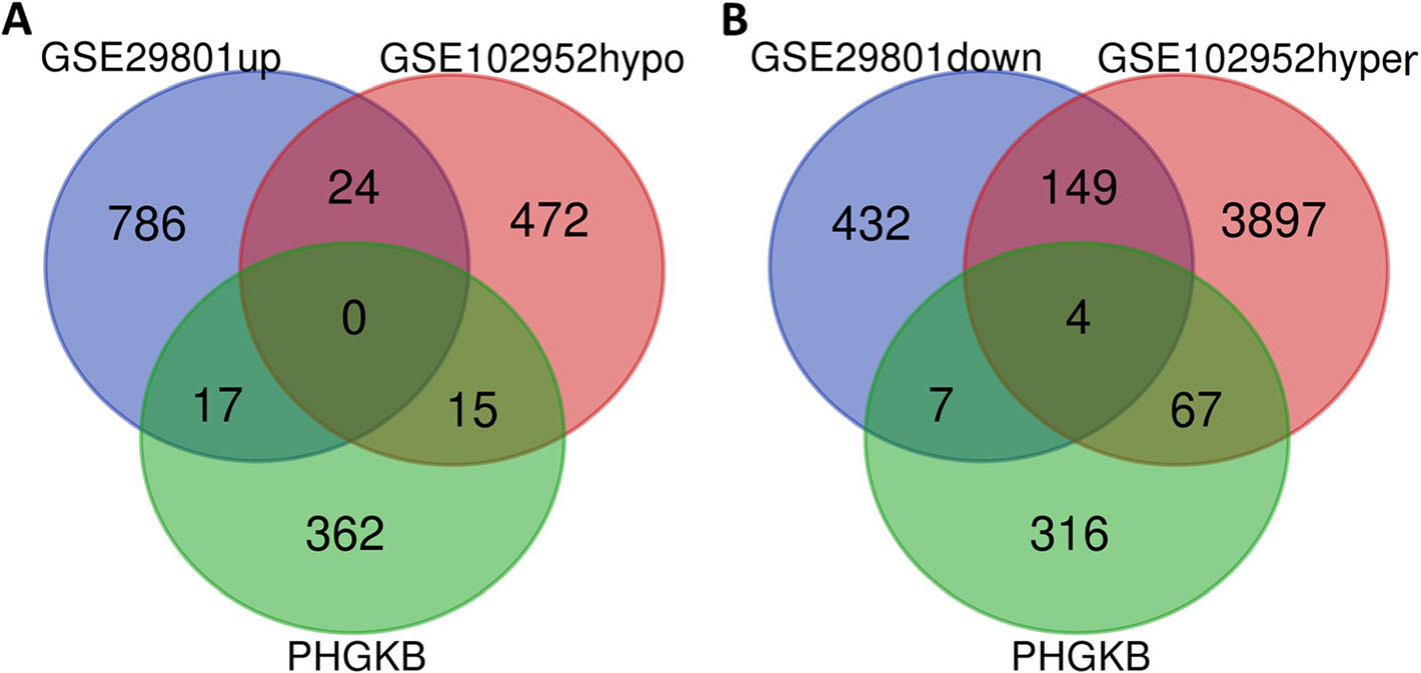
The results of overlapping GSE29801 and GSE102952 datasets with the genes reported to be related to AMD in Public Health Genomics and Precision Health Knowledge Base (PHGKB). **a** Overlapping of hypomethylation-high expression genes (Hypo-HGs) with the genes reported to be related to AMD in PHGKB. **b** Overlapping of hypermethylation-low expression genes (Hyper-LGs) with the genes reported to be related to AMD in PHGKB

To further explore the aberrantly-methylated DEGs, Hypo-HGs were obtained by overlapping hypomethylated and upregulated genes, whereas Hyper-LGs were obtained by overlapping hypermethylated and downregulated genes. We identified 24 Hypo-HGs and 153 Hyper-LGs. The flowchart of this study is presented in Fig. 1. All genes are shown in Table S3. To confirm the reliability of the results, we compared aberrantly MDEGs from the GSE29801 and GSE102952 datasets with genes in the Public Health Genomics and Precision Health Knowledge Base (PHGKB; version. 5.8) (https://phgkb.cdc.gov/PHGKB/startPagePhenoPedia.action) studied in the category of “macular degeneration”; the results are shown in Fig. 2. We screened four Hyper-LGs (*CKB*, *PPP3CA*, *TGFB2*, and *SOCS2*) that overlapped with potential AMD risk genes in the PHGKB. However, no Hypo-HG overlapped in the PHGKB.

### Functional enrichment analysis

Functional enrichment analysis was conducted using the R package “clusterProfiler”, and all significantly enriched KEGG pathways associated with the 24 Hypo-HGs and 153 Hyper-LGs are shown in Table 1. The results of KEGG pathway enrichment analysis indicated that Hypo-HGs were significantly enriched in the phosphatidylinositol signaling system and calcium signaling pathway, whereas Hyper-LGs were significantly enriched in amphetamine addiction, morphine addiction, and sphingolipid metabolism. The top five GO terms in each category in which the 24 Hypo-HGs and 153 Hyper-LGs were significantly involved are shown in Tables 2 and 3, respectively. Functional enrichment analysis suggested that the 24 Hypo-HGs were enriched in the BP of fibroblast migration and positive regulation of neurological system process. The GO CC category revealed enrichment in membrane raft and membrane microdomain. The MF category showed enrichment in factors involved in neuropeptide receptor activity and coenzyme binding (Table 2). The 153 Hyper-LGs were enriched in the BP category of mRNA transport, among others. The CC category revealed enrichment in nuclear speck and neuronal cell body, among others. The MF category indicated enrichment in DNA binding and phosphoric ester hydrolase activity (Table 3).

**Table 1:** Kyoto encyclopedia of genes and genomes pathway analysis of aberrantly methylated differentially expressed genes in age-related macular degeneration (AMD)

Category	ID	Description	GeneRatio	BgRatio	*P* value	Q value	geneSymbol	Count
**Hypomethylated with high expression**								
KEGG_PATHWAY	hsa04070	Phosphatidylinositol signaling system	2/13	99/7866	0.01117	0.28231	PIP5K1A/PIP4P2	2
KEGG_PATHWAY	hsa04020	Calcium signaling pathway	2/13	193/7866	0.03911	0.31445	NTSR1/TACR1	2
**Hypermethylated with low expression**								
KEGG_PATHWAY	hsa05031	Amphetamine addiction	3/59	68/7866	0.01423	0.73750	GRIN2C/PPP3CA/STX1A	3
KEGG_PATHWAY	hsa05032	Morphine addiction	3/59	91/7866	0.03057	0.73750	GRK4/PDE10A/PDE4D	3
KEGG_PATHWAY	hsa00600	Sphingolipid metabolism	2/59	47/7866	0.04820	0.73750	PLPP2/SGPL1	2

Table 1. Kyoto Encyclopedia of Genes and Genomes (KEGG) pathway enrichment analyses were performed for the selected genes. All significantly enriched KEGG pathways with the 24 hypo-methylated, high-expression genes (Hypo-HGs) and 153 hyper-methylated, low-expression genes (Hyper-LGs) are shown. The cut-off criterion was *P* < 0.05. The results indicated that Hypo-HGs were significantly enriched in the phosphatidylinositol signaling system and calcium signaling pathway, whereas Hyper-LGs were significantly enriched in amphetamine addiction, morphine addiction, and sphingolipid metabolism

**Table 2:** Gene Ontology analysis of hypomethylated with high expression genes in age-related macular degeneration (AMD)

Category	Term	Description	GeneRatio	BgRatio	*P* value	Q value	GeneSymbol	Count
**Hypomethylated with high expression**								
GOTERM_BP	GO:0010761	fibroblast migration	2/22	41/18493	0.00108	0.11666	DDR2/PIP5K1A	2
GOTERM_BP	GO:0031646	positive regulation of neurological system process	2/22	58/18493	0.00214	0.11666	NTSR1/TNR	2
GOTERM_BP	GO:1901616	organic hydroxy compound catabolic process	2/22	72/18493	0.00328	0.11666	LDHD/NTSR1	2
GOTERM_BP	GO:0003333	amino acid transmembrane transport	2/22	86/18493	0.00465	0.11666	NTSR1/SLC7A4	2
GOTERM_BP	GO:0031644	regulation of neurological system process	2/22	127/18493	0.00988	0.11666	NTSR1/TNR	2
GOTERM_CC	GO:0045121	membrane raft	3/23	304/19659	0.00516	0.13697	NTSR1/SKAP1/TNR	3
GOTERM_CC	GO:0098857	membrane microdomain	3/23	305/19659	0.00520	0.13697	NTSR1/SKAP1/TNR	3
GOTERM_CC	GO:0098589	membrane region	3/23	316/19659	0.00574	0.13697	NTSR1/SKAP1/TNR	3
GOTERM_CC	GO:0032280	symmetric synapse	1/23	11/19659	0.01280	0.16553	NTSR1	1
GOTERM_CC	GO:0061827	sperm head	1/23	11/19659	0.01280	0.16553	TACR1	1
GOTERM_MF	GO:0008188	neuropeptide receptor activity	2/20	50/17632	0.00145	0.07933	NTSR1/TACR1	2
GOTERM_MF	GO:0050662	coenzyme binding	3/20	285/17632	0.00389	0.09513	GCAT/LDHD/UXS1	3
GOTERM_MF	GO:0034596	phosphatidylinositol phosphate 4-phosphatase activity	1/20	10/17632	0.01129	0.09513	PIP4P2	1
GOTERM_MF	GO:0008528	G protein-coupled peptide receptor activity	2/20	145/17632	0.01158	0.09513	NTSR1/TACR1	2
GOTERM_MF	GO:0015174	basic amino acid transmembrane transporter activity	1/20	11/17632	0.01241	0.09513	SLC7A4	1

Table 2. Gene ontology (GO) pathway enrichment analyses were performed for the selected genes. The cut-off criterion was *P* < 0.05. The top 5 GO terms in each category in which the 24 hypo-methylated, high-expression genes were significantly involved are shown. They were enriched in the biological process of fibroblast migration and positive regulation of neurological system process. The cellular component category revealed enrichment in membrane raft and membrane microdomain. The molecular function category showed enrichment for factors involved in neuropeptide receptor activity and coenzyme binding

**Table 3:** Gene Ontology analysis of hypermethylated with low expression in age-related macular degeneration (AMD)

Category	Term	Description	GeneRatio	BgRatio	*P* value	Q value	GeneSymbol	Count
**Hypermethylated with low expression**								
GOTERM_BP	GO:0051028	mRNA transport	7/141	151/18493	0.00016	0.11773	DHX9/HNRNPA3/NUP58/SLU7/SMG1/SRSF11/YTHDC1	7
GOTERM_BP	GO:0042698	ovulation cycle	5/141	68/18493	0.00017	0.11773	ADAMTS1/ADNP/PAM/SGPL1/TGFB2	5
GOTERM_BP	GO:0022602	ovulation cycle process	4/141	47/18493	0.00045	0.11773	ADAMTS1/PAM/SGPL1/TGFB2	4
GOTERM_BP	GO:0060021	roof of mouth development	5/141	89/18493	0.00060	0.11773	CHD7/FOXE1/MEF2C/SGPL1/TGFB2	5
GOTERM_BP	GO:0021513	spinal cord dorsal/ventral patterning	3/141	22/18493	0.00060	0.11773	INTU/PAX6/SOX1	3
GOTERM_CC	GO:0016607	nuclear speck	12/146	382/19659	0.00003	0.00790	BAZ2A/DPP3/FAM76B/MAPT/MEF2C/PNISR/SLU7/SREK1/SRSF11/TARDBP/WAC/YTHDC1	12
GOTERM_CC	GO:0043025	neuronal cell body	12/146	483/19659	0.00027	0.03616	ADNP/CCK/CKB/GRK4/KCND3/MAPT/MPL/PAM/PDE10A/PTPRF/RTN4RL1/TGFB2	12
GOTERM_CC	GO:0044441	ciliary part	9/146	441/19659	0.00575	0.36035	AHI1/CEP126/CEP131/DYNC2H1/GRK4/INTU/MLF1/NEK8/NIN	9
GOTERM_CC	GO:0001669	acrosomal vesicle	4/146	106/19659	0.00808	0.36035	CEP131/FLOT2/SPAG8/SV2B	4
GOTERM_CC	GO:1990351	transporter complex	7/146	333/19659	0.01232	0.36035	ABCD4/GRIK1/GRIN2C/KCND3/PDE4D/PEX13/STX1A	7
GOTERM_MF	GO:0003680	AT DNA binding	2/141	10/17632	0.00274	0.26873	MAPT/MEF2C	2
GOTERM_MF	GO:0042578	phosphoric ester hydrolase activity	9/141	369/17632	0.00290	0.26873	CTDSP1/PDE10A/PDE4D/PDPR/PFKFB2/PLPP2/PPP3CA/PTPDC1/PTPRF	9
GOTERM_MF	GO:0044325	ion channel binding	5/141	125/17632	0.00332	0.26873	KCND3/PDE4D/SLC8A1/STX1A/YWHAZ	5
GOTERM_MF	GO:0001046	core promoter sequence-specific DNA binding	3/141	38/17632	0.00344	0.26873	BAZ2A/H3F3A/PAX6	3
GOTERM_MF	GO:0004721	phosphoprotein phosphatase activity	6/141	182/17632	0.00346	0.2687	CTDSP1/PDPR/PLPP2/PPP3CA/PTPDC1/PTPRF	6

Table 3. Gene ontology (GO) pathway enrichment analyses were performed for the selected genes. The cut-off criterion was *P* < 0.05. The top 5 GO terms in each category in which the 153 hyper-methylated, low-expression genes were significantly involved are shown. They were enriched in the biological process of mRNA transport, etc. The cellular component category revealed enrichment in nuclear speck, neuronal cell body, etc. The molecular function category indicated enrichment in DNA binding and phosphoric ester hydrolase activity

### Comprehensive gene regulation network

**Fig. 3:**
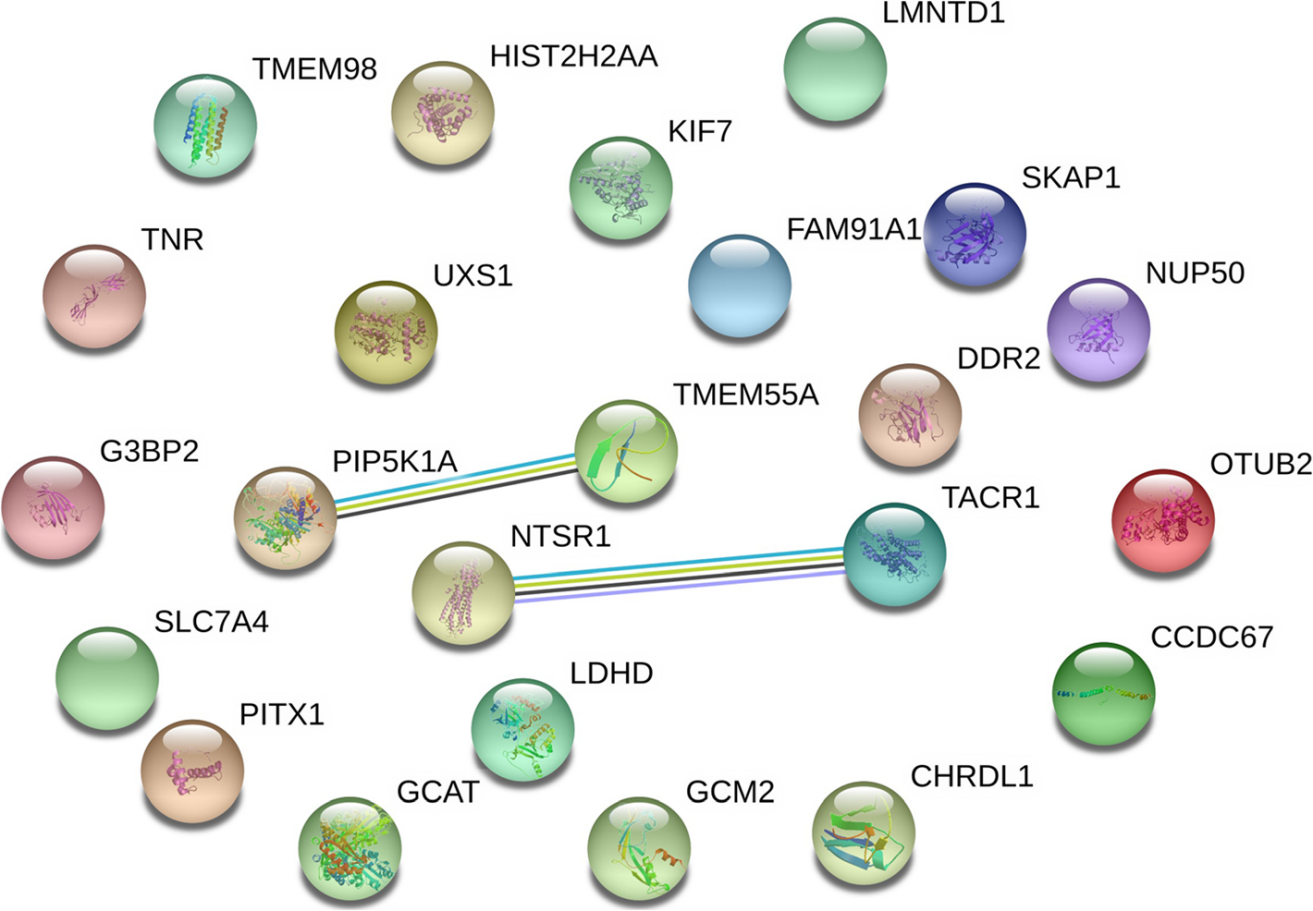
The protein–protein interaction (PPI) networks of the 24 hypomethylation-high expression genes (Hypo-HGs). 23 nodes and 2 edges were established from the Hypo-HGs

**Fig. 4:**
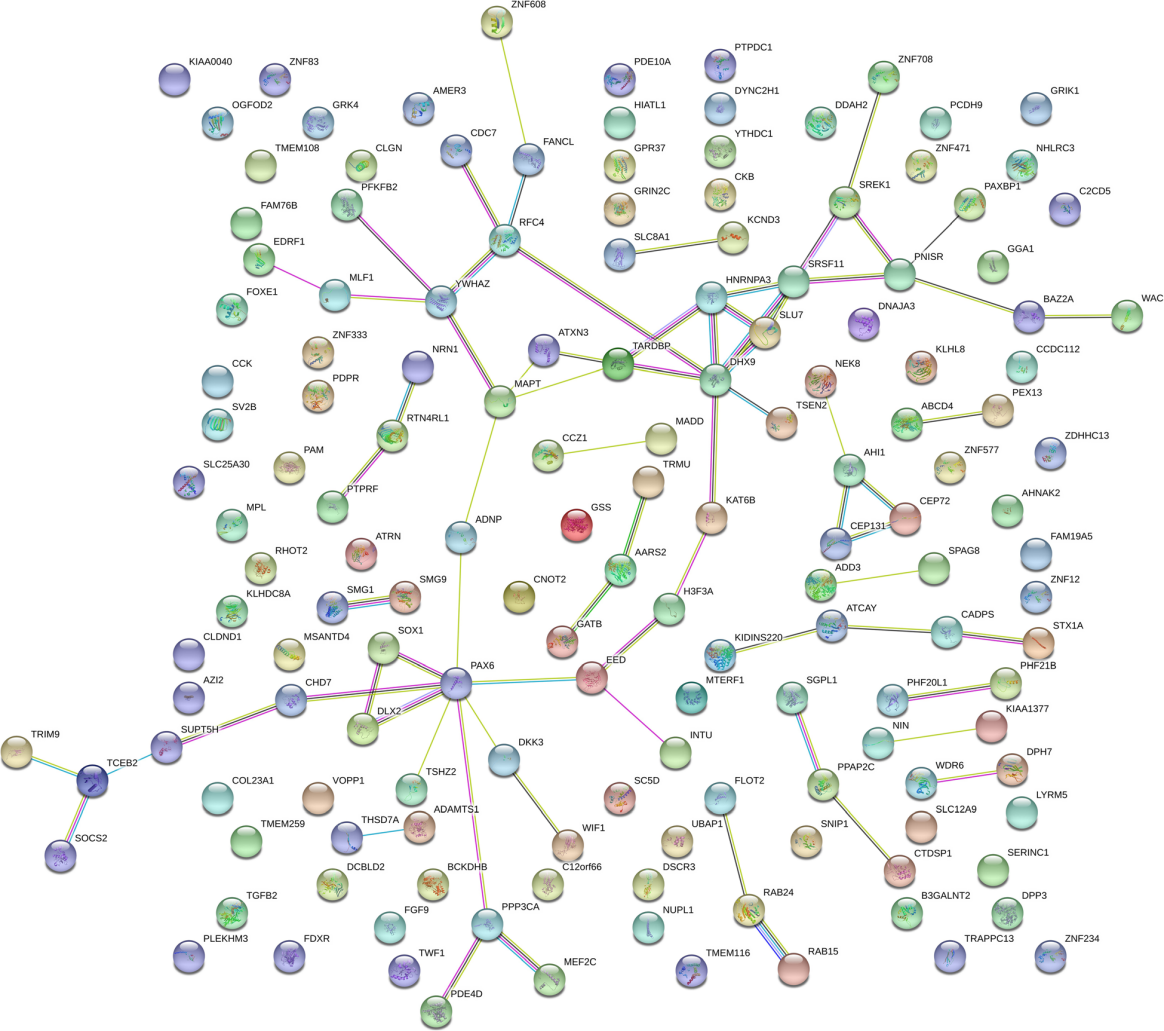
The protein–protein interaction (PPI) networks of the 153 hypermethylation-low expression genes (Hyper-LGs). 151 nodes and 73 edges were established from the Hyper-LGs

**Fig. 5:**
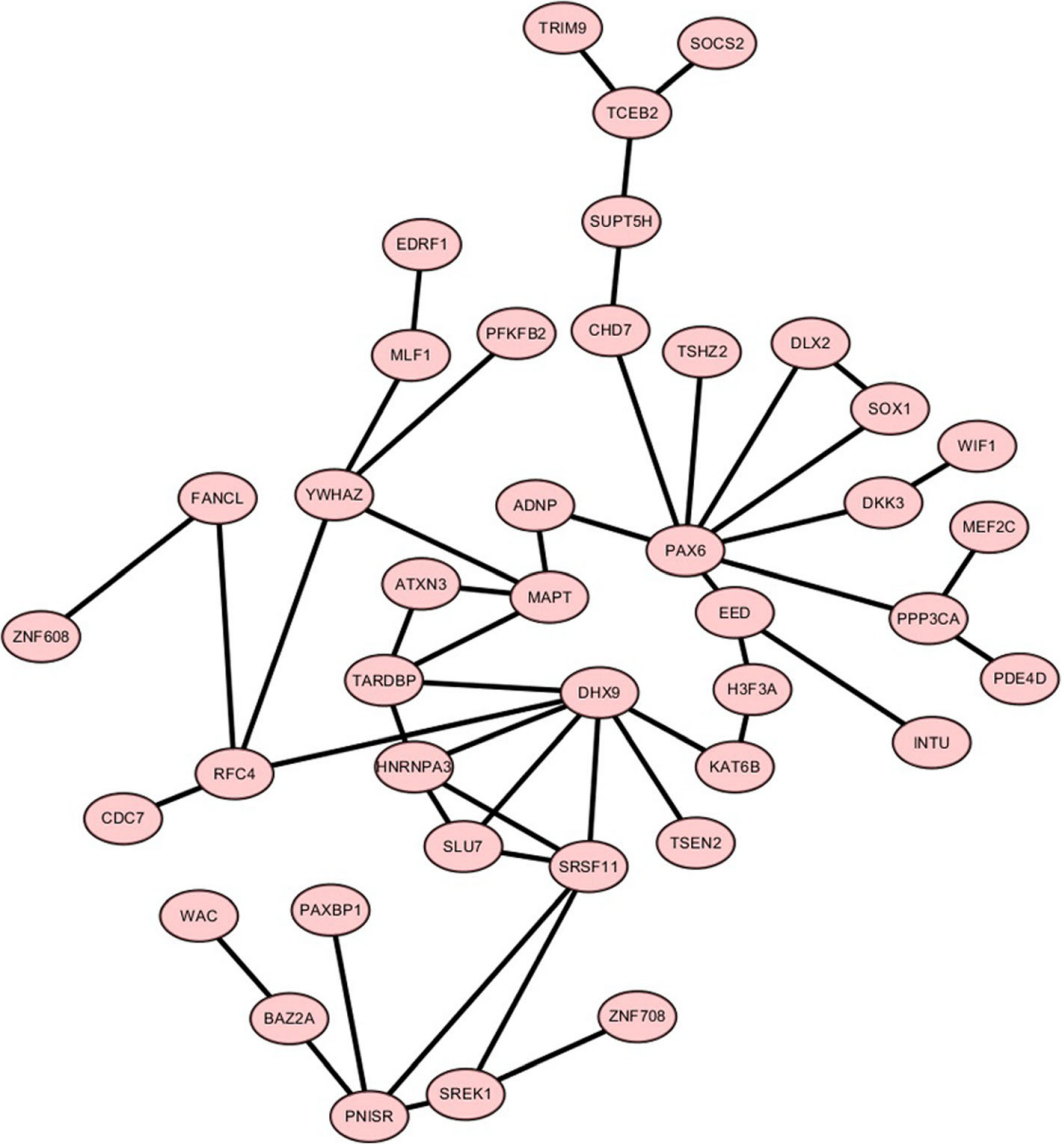
The subgroup protein–protein interaction (PPI) analysis of the hypermethylation-low expression genes (Hyper-LGs)

The STRING database was used to construct PPI networks. Ultimately, 23 nodes and two edges were established from the Hypo-HGs and 151 nodes and 73 edges were established from the Hyper-LGs. The results are shown in Figs. 3 and 4. We use Cytoscape software to determine the largest subgroup interaction network of Hyper-LG genes (Fig. 5). We identified hub genes using cytoHubba and confirmed that *DHX9*, *MAPT*, and *PAX6* showed the greatest overlap. Two-core module analysis of this subgroup network of Hyper-LG genes was performed, including module1 comprising *HNRNPA3*, *DHX9*, *SRSF11*, and *SLU7* and module2 comprising *SOX1*, *PAX6*, and *DLX2*.

## Discussion

AMD is a disease with complex inheritance and epigenetic changes [5]. Identification of the underlying genes has been difficult. Both genomic screening (locational) and candidate gene (functional) approaches have been used. Based on numerous genetic studies of AMD, approximately 50% of the heritability of AMD can be explained by two major loci harboring coding and non-coding variations at chromosomes 1q (*CFH*) and 10q (*ARMS2*/*HTRA1*) [25–28]. Recently, a large GWAS highlighted new genes and pathways involved in the development of AMD, including complement activation, collagen synthesis, lipid metabolism/cholesterol transport, receptor-mediated endocytosis, endodermal cell differentiation, and extracellular matrix organization, indicating that many unknown genetic changes remain to be identified with respect to the initiation and development of AMD [20]. The application of novel drugs in the treatment of macular disease also indicated the complicated change of the micro-environment of the macular in the case of disease [29–31]. In this study, we screened novel biomarkers by combining microarray information from RPE-choroid and retinal tissue samples from patients with AMD, as well as peripheral blood samples, by overlapping relevant datasets (GSE29801 and GSE10295) using integrated bioinformatics analysis for available microarray data. This is the first study to employ this approach.

The Hyper-LGs identified are potential biomarkers of AMD based on methylation microarrays for pre-clinical detection in peripheral blood. Among them, four Hyper-LGs (*CKB*, *PPP3CA*, *TGFB2*, and *SOCS2*) overlapped with risk genes in the category of “macular degeneration” in the PHGKB. One study revealed that *CKB* is unlikely to explain a significant portion of the risk of developing AMD in a family-based association dataset including 162 families and an independent case-control dataset of 399 cases and 159 fully evaluated controls [32]. *PPP3CA* is a druggable molecule that inactivates MAP3K5 but has not been widely investigated for its role in AMD. One previous study revealed AMD-related sequence variants in genes encoding *PPP3CA*, underlying its relationship with AMD [33]. *TGFB2* induces RPE cell and collagen gel contraction. Subretinal fibrosis contributes to the loss of vision associated with AMD, and RPE cells play a key role in the fibrotic reaction [34]. Under hypoxic conditions, RPE cells can increase the secretion of *TGFB2* and induce epithelial–mesenchymal transition, resulting in the formation of scar-like fibrous tissue in AMD [35]. Targeted inhibition of *TGFB* signaling might be an effective approach to retard AMD progression [36]. SOCS proteins are modulators of cytokine and growth factor signaling, and their aberrant regulation has been linked to a variety of inflammatory and neoplastic diseases [37]. In a GWAS of 919 patients with exudative AMD treated with intravitreal ranibizumab, *SOCS2* was a candidate gene for which levels were associated with visual loss at month three [38]. These results provide insight into AMD pathogenesis but must be confirmed by in vivo and in vitro experiments. The methylation patterns of *PPP3CA*, *TGFB2*, and *SOCS2* in AMD have not been previously described. We found that these genes were hypermethylated and expressed at low levels, suggesting that the aberrant methylation of these genes affects the pathogenesis of AMD. No Hypo-HGs overlapped in the PHGKB, likely because of the limited number of genes identified.

Among the top five pathways identified by KEGG and GO analyses, calcium signaling [39, 40], sphingolipid metabolism [41, 42], fibroblast migration [43, 44], membrane [45], coenzyme [46–48], and DNA binding [49] have been investigated in AMD. Calcium signaling, sphingolipid metabolism, and coenzyme categories showed strong relationships with AMD, whereas the others require further evaluation. Calcium signaling plays a vital role in RPE cell function. Intracellular calcium mobilization activates gene expression and the secretion of inflammatory cytokines such as interleukin-8 in human RPE cells [39]. Complement attack on RPE cells, leading to cell death, is also modulated by extracellular calcium and intracellular signaling mechanisms [40]. Sphingosine 1-phosphate is a potent lipid mediator that modulates inflammatory responses and proangiogenic factors, and it has been suggested that this protein upregulates CNV and is deeply involved in the pathogenesis of exudative AMD [42]. Free radicals play a pathogenic role in AMD, whereas coenzyme Q10 has a protective effect [48]. A combination of acetyl-L-carnitine, n-3 fatty acids, and coenzyme Q10 was shown to be beneficial for visual functions in early AMD [47]. However, drug metabolism pathways such as amphetamine addiction and morphine addiction could have been identified by chance and might not be related to AMD. The specific manner in which the other pathways affect AMD development and progression must be further investigated.

In the PPI network, 23 nodes and two edges were established from the Hypo-HGs and 151 nodes and 73 edges were established from the Hyper-LGs. *DHX9*, *MAPT*, and *PAX6* were identified as hub genes. Two core modules for Hyper-LGs were structured, including module1 comprising *HNRNPA3*, *DHX9*, *SRSF11*, and *SLU7* and module2 comprising *SOX1*, *PAX6*, and *DLX2*. Among the hub genes and core modules previously mentioned, *PAX6* is expressed in retinal progenitor cells throughout retinogenesis [50]. *PAX6* is a novel regulatory gene among RPE transcription factors that controls the timing of RPE differentiation and adjacent choroid maturation, suggesting that *PAX6* is involved in choroid development during the pathogenesis of AMD [51]. Other genes have not been previously investigated with respect to AMD.

This study aimed to find potential biomarkers of AMD based on public datasets and bioinformatics methods. However, the results of this study were not strong enough to switch the diagnosis and treatment of AMD so far. These years, ophthalmology has experienced significant developments with respect to imaging modalities. Optical coherence tomography (OCT) is a non-invasive imaging modality that produces high-resolution, cross-sectional images of ocular tissues. Compared to time-domain OCT, spectral-domain OCT yields a higher degree of axial resolution and provides more detailed views of intraretinal structure [52]. Swept-source OCT can offer improved images of the choroid and pigmented lesions [53]. The development of OCT benefits to the diagnosis and follow-up of AMD, and we guess the early detection based on MDEGs might help to identify AMD patients before the clinical symptoms appear. It might be possible to develop detection reagents in the blood for early detection and screening of AMD in the future.

There were some limitations of this study. First, we focused on Hyper-LGs and Hypo-HGs without analyzing contra-regulated genes; thus, further analysis is required to evaluate these genes. Second, our study was limited to only two datasets, and we did not conduct validation based on animals or patient samples. Thus, the results are preliminary and larger sample sizes as well as further fundamental experiments are needed to confirm these results. Third, the clinical characteristics of AMD patients included were not analyzed because these data were not available, and thus, the results should be conservatively interpreted.

## Conclusions

In summary, data from gene expression profiling microarrays and gene methylation profiling microarrays of patients with AMD were integrated and analyzed using a series of bioinformatics tools. Our results indicated aberrantly MDEGs (*PPP3CA*, *TGFB2*, and *SOCS2*) and pathways (calcium signaling, sphingolipid metabolism, fibroblast migration, membrane, coenzyme, and DNA binding) associated with AMD. These genes might serve as biomarkers for the precise diagnosis and treatment of AMD. Further studies are needed to confirm the functional significance of the identified genes and pathways in AMD.

## Supplementary information

**Additional file 1: Table S1.** The top 100 most significantly differentially expressed genes (DEGs) of GSE29801. To identify DEGs, we used the expression profile from GSE29801 (containing RPE-choroid and retina tissue samples from 41 patients with AMD and 42 normal samples). After data preprocessing and quality assessment using R software, we identified 827 high-expression genes and 592 low-expression genes. The top 100 most significantly DEGs of GSE29801 are shown in Table S1.

**Additional file 2: Table S2.** The top 100 most significantly differentially methylated genes (DMGs) of GSE29801. To identify DMGs, we used the methylation profile from GSE102952 (containing peripheral blood samples of 9 AMD patients and 9 normal controls). After data preprocessing and quality assessment using R software, we identified 4117 hypermethylated genes and 511 hypomethylated genes. The top 100 most significantly DMGs of GSE102952 are shown in Table S2.

**Additional file 3: Table S3**. Identification of aberrantly methylated-differentially expressed genes (DEGs) in age-related macular degeneration (AMD). To further explore the aberrantly methylated DEGs, hypo-methylated, high-expression genes (Hypo-HGs) were obtained by overlapping hypomethylated and up-regulated genes; hyper-methylated, low-expression genes (Hyper-LGs) were obtained by overlapping hypermethylated and down-regulated genes. We identified 24 Hypo-HGs and 153 Hyper-LGs, and all genes are shown in Table S3.

**Additional file 4..** The statement provided by the ethics committee of Shanghai General Hospital, Shanghai Jiao Tong University School of Medicine, Shanghai, China.

## Data Availability

The datasets analysed during the current study are available in the Gene Expression Omnibus (GEO, https://www.ncbi. nlm.nih.gov/geo/) of the National Center for Biotechnology Information (NCBI). (GSE29801: https://www.ncbi.nlm.nih.gov/geo/query/acc.cgi?acc=GSE29801; GSE102952: https://www.ncbi.nlm.nih.gov/geo/query/acc.cgi?acc=GSE102952).

## Abbreviations

AMD: Age-related macular degeneration
BP: Biological process
CC: Cellular component
CNV: Choroidal neovascularization
DEGs: Differentially expressed genes
DMGs: Differentially methylated genes
GEO: Gene Expression Omnibus
GO: Gene ontology
GWASs: Genome-wide association studies
Hyper-LGs: Hyper-methylated low-expression genes
Hypo-HGs: Hypo-methylated high-expression genes
KEGG: Kyoto Encyclopedia of Genes and Genomes
MDEGs: Methylated-differentially expressed genes
MF: Molecular function
NCBI: National Center for Biotechnology Information
PHGKB: Public Health Genomics and Precision Health Knowledge Base
PPI: Protein–protein interaction
RPE: Retinal pigment epithelium
STRING: Search Tool for the Retrieval of Interacting Genes
