# Supplementary material for: Integrated bioinformatics analysis of aberrantly-methylated differentially-expressed genes and pathways in age-related macular degeneration
Source: BMC Ophthalmol. 2020 Mar 24;20:119. doi: 10.1186/s12886-020-01392-2 (PMC7092446; doi:10.1186/s12886-020-01392-2)
Supplement: Supplementary file 4 — Additional file 4.. The statement provided by the ethics committee of Shanghai General Hospital, Shanghai Jiao Tong University School of Medicine, Shanghai, China. [file 12886_2020_1392_MOESM4_ESM.pdf]

**The statement provided by the ethics committee of Shanghai General Hospital, Shanghai Jiao Tong University School of Medicine, Shanghai, China.**

说明

因《表达谱芯片与 DNA 甲基化芯片综合分析发现年龄相关性黄斑变性患者发生、发展的分子靶标》不涉及人体试验伦理审查，故不出具伦理批件，特此说明。

上海市第一人民医院

人体试验伦理委员会

**Translation**

**Statement**

**There is no need to issue an ethical approval document for the study named "Integrated bioinformatics analysis of aberrantly methylated-differentially expressed genes and pathways in age-related macular degeneration" since it does not involve the ethical review of human experiments.**

**The ethics committee of Shanghai General Hospital,**
**Shanghai Jiao Tong University School of Medicine, Shanghai, China**
